# Bacterial diversity in *Haemagogus leucocelaenus* (Diptera: Culicidae) from Vale do Ribeira, São Paulo, Brazil

**DOI:** 10.1186/s12866-022-02571-5

**Published:** 2022-06-22

**Authors:** Herculano da Silva, Tatiane M. P. Oliveira, Ester C. Sabino, Diego Peres Alonso, Maria Anice M. Sallum

**Affiliations:** 1 Departamento de Epidemiologia, Faculdade de Saúde Pública, Universidade de São Paulo, Av. Dr. Arnaldo 715, São Paulo, SP 01246-904 Brazil; 2 Departamento de Moléstias Infecciosas e Parasitarias, Instituto de Medicina Tropical da Faculdade de Medicina da Universidade de São Paulo, Av. Dr. Eneas de Carvalho 470, 1º andar, São Paulo, 05403-000 Brazil; 3 Biotechnology Institute and Bioscience Institute, Sao Paulo State University (UNESP), Botucatu, 18618-689 Brazil

**Keywords:** Mosquito vector, Yellow Fever, Microbiota, Atlantic Forest

## Abstract

**Introduction:**

Mosquitoes (Diptera: Culicidae) are vectors that transmit numerous pathogens to humans and other vertebrates. *Haemagogus leucocelaenus* is a mosquito associated with transmission of yellow fever virus. The insect gut harbors a variety of microorganisms that can live and multiply within it, thus contributing to digestion, nutrition, and development of its host. The composition of bacterial communities in mosquitoes can be influenced by both biotic and abiotic factors. The goal of this study was to investigate the bacterial diversity of *Hg. leucocelaenus* and verify the differences between the bacterial communities in *Hg. leucocelaenus* from three different locations in the Atlantic tropical rain forest and southeastern state of São Paulo State, Brazil.

**Results:**

The phylum *Proteobacteria* was found in mosquitoes collected from the three selected study sites. More than 50% of the contigs belong to *Wolbachia*, followed by 5% *Swaminathania*, and 3% *Acinetobacter*. The genus *Serratia* was found in samples from two locations.

**Conclusions:**

*Wolbachia* was reported for the first time in this species and may indicates that the vector competence of the populations of the species can vary along its geographical distribution area. The presence of *Serratia* might facilitate viral invasion caused by the disruption of the midgut barrier via action of the SmEnhancin protein, which digests the mucins present in the intestinal epithelium.

**Supplementary Information:**

The online version contains supplementary material available at 10.1186/s12866-022-02571-5.

## Background

Approximately 3,500 species of mosquitoes (Diptera: Culicidae) have been found worldwide, and several species are known to be vectors of several pathogens. Mosquitoes harbor diverse and dynamic bacterial communities mainly in their midguts, salivary glands, body external surfaces, and reproductive organs [1, 2]. Symbiotic bacteria of the microbiota play a key role in both the biological development of mosquitoes and modulating pathogen infections in these insects [3–5]. For instance, *Serratia marcescens* Y1 strain renders *Anopheles sthephensi* resistant to *Plasmodium berghei* infection [6]. In addition, *Wolbachia* wMelPop and wAlbB strain infections were found to cause significant inhibition of the development of *Plasmodium falciparum* oocyst in the midgut of *An. gambiae* [7] and lead to a reduction in the vector competence of a population infected. Also, *Wolbachia* infection inhibited replication of the yellow fever and Chikungunha viruses in *Aedes aegypti* [8], process that could lead to mosquito population that is resistant to the Zika virus infection [9].

The composition of bacterial communities in mosquitoes can be influenced by both biotic and abiotic factors [10, 11] and vary between tissues, species, sex, geographic locations, and developmental stages [12–15]. In addition, vector-borne pathogen infections can affect the composition of the gut microbiota of mosquitoes [16–18]. The environment can play a key role in determining the bacterial composition in adult mosquitoes [19]. The environmental effect in the microbiota is associated with the exposure of these insects to the bacteria present in their habitats. Mosquito–environmental bacterial contact occurs during the developmental stages (transstadally) [20] and when the recently emerged adult takes in water from the breeding site [21]. In addition, blood feeding can influence the gut microbiota composition, thus affecting the diversity in mosquitoes [17, 22, 23].

The bacterial community differs in mosquitoes collected from different geographic locations [24]. The diversity of the microbiota and the relative abundance of bacterial families in *An. gambiae* and *An. coluzzii* mosquitoes differ in relation to geographical locality from which they were collected and the season [25]. In addition, Tchouassi and colleagues [26] showed that the collection site influences the richness, diversity, and bacterial composition in two vectors species of the Rift Valley fever virus in Kenya.

The *Haemagogus* genus consists of 28 species [27], several of which are of great public health importance because they are involved in the maintenance of several zoonoses sylvatic cycles [28]. *Hg. leucocelaenus* is an essentially sylvatic species whose main habitat is the tree canopy in which they develop their activities during the daytime [29, 30]. Its geographic distribution extends from Trinidad to southern Brazil and northern Argentina [31]. In Brazil, the species occurs in the South, Southeast, and Midwest states [32]. This species has received special attention due to its growing importance in public health because of its involvement in the recent epidemics of the yellow fever [33, 34].

*Hg. leucocelaenus* is considered the primary vector of sylvatic yellow fever virus in the south-eastern region of Brazil [35, 36]. Despite the public health importance of *Hg. leucocelaenus*, no studies in the literature have focused on the bacterial diversity and the influence of the environment in the composition of its microbiota. Overall, the aims of this study were to investigate the bacterial diversity of *Hg. leucocelaenus* and verify potential differences between the bacterial communities in *Hg. leucocelaenus* collected from three different sites in the Vale do Ribeira, São Paulo state, Brazil.

## Methods

### Study areas

**Fig. 1:**
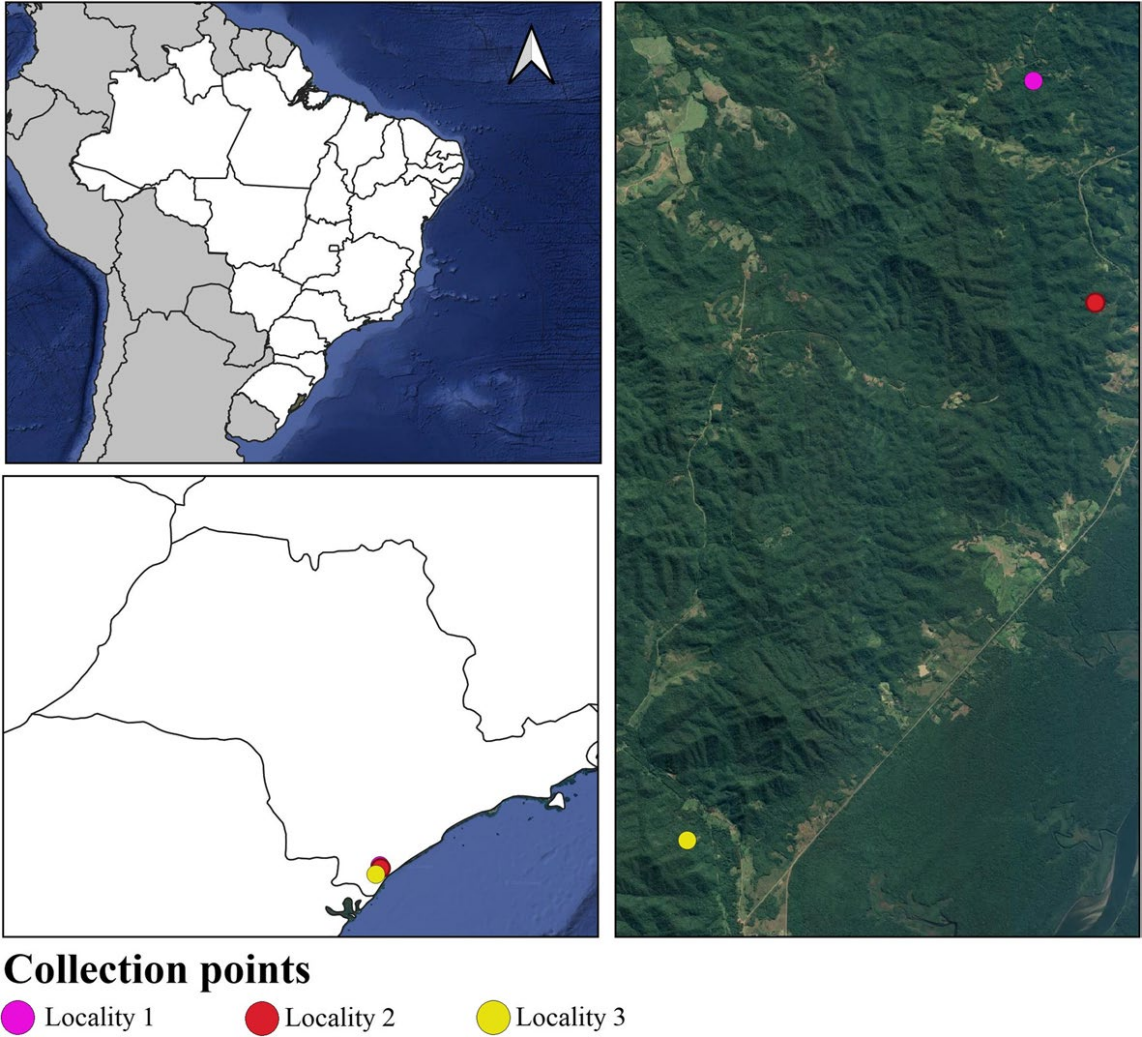
Map of sampling locations across the Vale do Ribeira, southeeastern São Paulo state, Brazil

This study was conducted at three collection sites in the Vale do Ribeira, São Paulo state, Brazil: (1) Locality #1, Sítio Francisco (24º47′28.8″ S, 47º54′42.6″ W), Pariquera-Açu municipality; (2) Locality #2, Sítio Rui (24º49′43.2″ S, 47º54′05.6″ W), Cananeia municipality; and (3) Locality #3, Sítio Alfredo (24º55′09.05″ S, 47º58′12.6″ W) Cananeia municipality (Fig. 1). The Vale do Ribeira Region comprises the largest continuous area of preserved Atlantic Tropical Rain Forest and has a great diversity of fauna and flora [37]. In addition, the region harbours several species of potential mosquito vectors [38–41], including the Rocio Virus [42], isolated from *Psorophora ferox* [43].

### Mosquito collection and species identification

Field collections were conducted from January to February 2020. Using an entomological net, adult mosquitoes were collected from 8:00 to 14:30 h daily during this period at the previously mentioned collection sites. Live mosquitoes were transported to the campus base of the University of São Paulo in Pariquera-Açu. Specimens were kept at temperature − 20 ºC for 20 to 30 min after which time they were morphologically identified using the identification key of Forattini [44]. Following identification, the mosquitoes were individually conditioned in ethanol 100 PA and preserved at − 20 ºC. Samples were transported on ice to the Laboratório de Entomologia em Saúde Pública–Sistemática Molecular and stored at − 70 ºC until processing.

### Sequencing of the V4 region of 16S rRNA

Genomic DNA extraction was performed individually from whole mosquitoes using Quick-DNA Fungal/Bacterial Miniprep kit (ZymoResearch) according to the manufacturer's instructions. Polymerase chain reaction (PCR) amplification of the V4 region of the 16S rRNA gene was performed with a final volume of 20 µL containing 1X GoTaq® Colorless Master Mix (Progema, USL), 0.3 µM of each primer (16S-V4 Forward: 5' TCGTCGGCAGCGTCAGATGTGTATAAGAGACAGGTGCCAGCMGCCGCGGTAA 3'; 16S- V4 Reverse: 5' GTCTCGTGGGCTCGGAGATGTGTATAAGAGACAGGGACTACHVGGGTWTCTAAT 3'), ultrapure water, and 1 µL of genomic DNA. The PCR thermal conditions were set to specific conditions: (1) 94 °C for 3 min followed by 30 cycles of 94 °C for 45 s, (2) 55 °C for 1 min, (3) 72 °C for 1 min and 30 s, and (4) a final extension of 72 °C for 10 min. The reactions were performed in a Veriti™ Thermal Cycler (Applied Biosystems). The PCR products were visualized in a 2% agarose gel stained with Unisafe Dye 0.03% (v/v) and purified with AgencourtAMPure XP magnetic beads (Beckman Coulter, Brea, USA) following the manufacturer's instructions. The Nextera XT Index kit (Illumina) was used for insertion of the indices according to the manufacturer’s protocol. After indexing, the products were purified with magnetic beads and then quantified by real-time PCR (qPCR) with KAPA-KK4824 kit (Library Quantification kit – Illumina/Universal) in QuantStudio 3 (Applied Biosystems) according to the manufacturer’s recommendations. All samples were normalized to 4 nM, and an equimolar pool of PCR amplicons was prepared. Next generation sequencing (NGS) was performed using the MiSeq Reagent Micro v2 kit (300 cycles) on Illumina MiSeq sequencer (Illumina, San Diego, USA).

### Processing of sequences and taxonomic attribution

Illumina paired-end reads were assembly in FLASH v. 1.2.11 [45] with minimmum overlap of six base pairs. The QIIME 2 v.2021–11 software [46] was used to process contigs and perform taxonomic attribution, diversity, and abundance analyses. The contigs sequences were imported into the QIIME2 via the *qiime tools import* command using Casava 1.8 single-end demultiplexed format. Quality control of the contigs and removal of chimeras were performed with Dada2 plugin of QIIME2 (q2-dada2 denoise). The Dada2 plugin generates a feature table of amplicon sequence variants (ASVs) instead of an operational taxonomic unit (OTU) table. For taxonomic attribution, a Greengenes 13_8 99% classifier and several commands were used: (1) *qiime feature-classifier classify-sklearn*, (2) *qiime metadata tabulate*, and (3) *qiime taxa barplot*.

### Data analyses

#### Rarefaction curve and diversity analysis

A rarefaction curve was constructed to investigate whether the richness of the samples was entirely distinguishable. This curve was generated using the command *qiime diversity alpha-rarefaction* and a depth value of 40,000. To obtain the diversity metrics, samples were normalized using the *qiime diversity core-metrics-phylogenetic* comand. A phylogenetic tree was generated with *qiime phylogeny align-to-tree-mafft-fasttree.*

The Shannon–Weaver indices (α diversity) were calculated to estimate bacterial diversity in each sample. These values were generated in QIIME 2 and subject to Kruskal–Wallis test, a nonparametric method, in RStudio v.1.4.1106 to verify whether samples from one collection site have greater α diversity than from other collection sites.

The β diversity index was obtained to determine whether bacterial diversity differs between mosquitoes collected from different collection sites. Theses indices were generated using the *qiime diversity beta-group-significance* command and Unifrac distances data (weighted and unweighted) generated in QIIME 2. The β diversity values were submitted to a permutational multivariate analysis of variance (PERMANOVA) statistical test to verify whether β diversity was significantly different between the groups.

### Differential abundance

Microbiome composition analysis (ANCOM) was used to verify the differential abundance of amplicon sequence variants (ASVs) in mosquitoes from different collection sites. The ANCOM was performed in QIIME using Greengenes taxonomy data and several commands: (1) *qiime taxa collapse*, (2) *qiime composition add-pseudocount* and (3) *qiime composition ancom*.

### Principal coordinate analysis and heatmap

Principal coordinate analysis (PCoA) was performed to visualize the distance between bacterial communities (β diversity) of the samples obtained from different collection sites according to phylogenetic information. The weighted and unweighted Unifrac phylogenetic distance matrices were used to generate PCoAs. The unweighted Unifrac is a qualitative measure and uses only taxon presence or absence, while the weighted Unifrac is quantitative. The latter considers taxon relative abundance in addition to the presence or absence of it in the microbiota. The Unifrac distance matrices were generated in QIIME2, and the PCoA images were analysed in RStudio v.1.4.1106. Heatmaps were generated to visualize 16S metagenomic profile of the abundance relative to the bacterial families and genera present in each sample. The heatmaps were carried out in RStudio v.1.4.1106 employing QIIME 2 output files.

## Results

### Sequences data

Twenty-nine *Hg. leucocelaenus* females (Supplementary Table 1) were used to generate the bacterial 16S rRNA dataset. NGS generated a total of 2,845,511 (R1 and R2) raw reads. The number of reads obtained per sample varied between 79,442 (R1 and R2) and 205,786 (R1 and R2) as shown in Supplementary Table 2. After assembly and de-noising, a total of 2,442,247 contigs were retained for analyses (Supplementary Table 2).

### Bacterial diversity

**Fig. 2:**
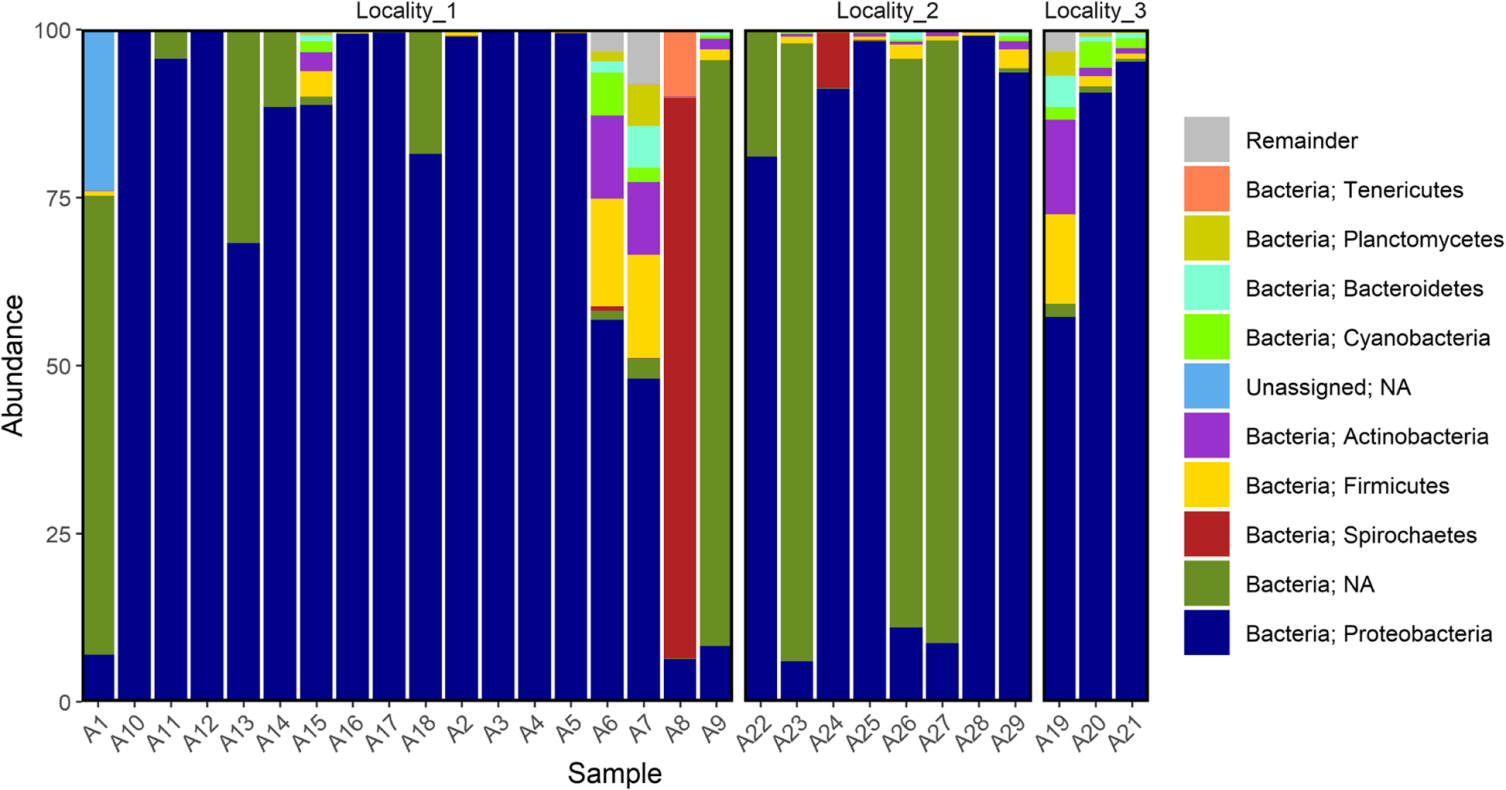
Phylum-level taxonomic amplicon sequence variants (ASV) composition in each female sampled

**Fig. 3:**
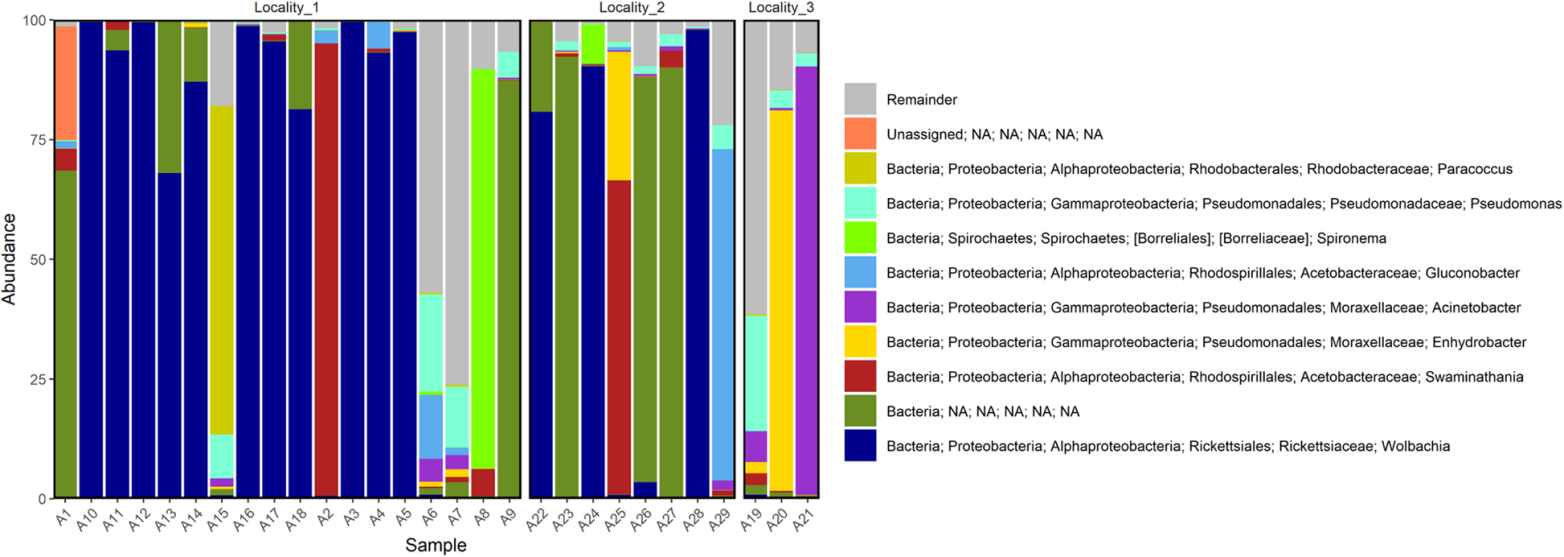
Genus-level taxonomic amplicon sequence variants (ASV) composition in each female sampled

A total of 613 ASVs were identified in samples collected from Locality #1, 384 from Locality #3, and 345 from Locality #2 (Supplementary Table 3). *Proteobacteria* was the predominant phylum, and bacteria from this phylum were found in all samples (Fig. 2). The abundance of *Wolbachia* genus varied between samples (Fig. 3), and only one female did not present any evidence of this bacteria genus (Supplementary Table 3). More than 50% of the contigs were identified as *Wolbachia* followed by the *Swaminathania* and *Acinetobacter* genera (5% and 3%, respectively) (Supplementary Table 3). *Serratia marcescens* was found in all samples collected from Locality #3 and in two females from Locality #1 (Supplementary Table 3).

### Diversity analysis

**Fig. 4:**
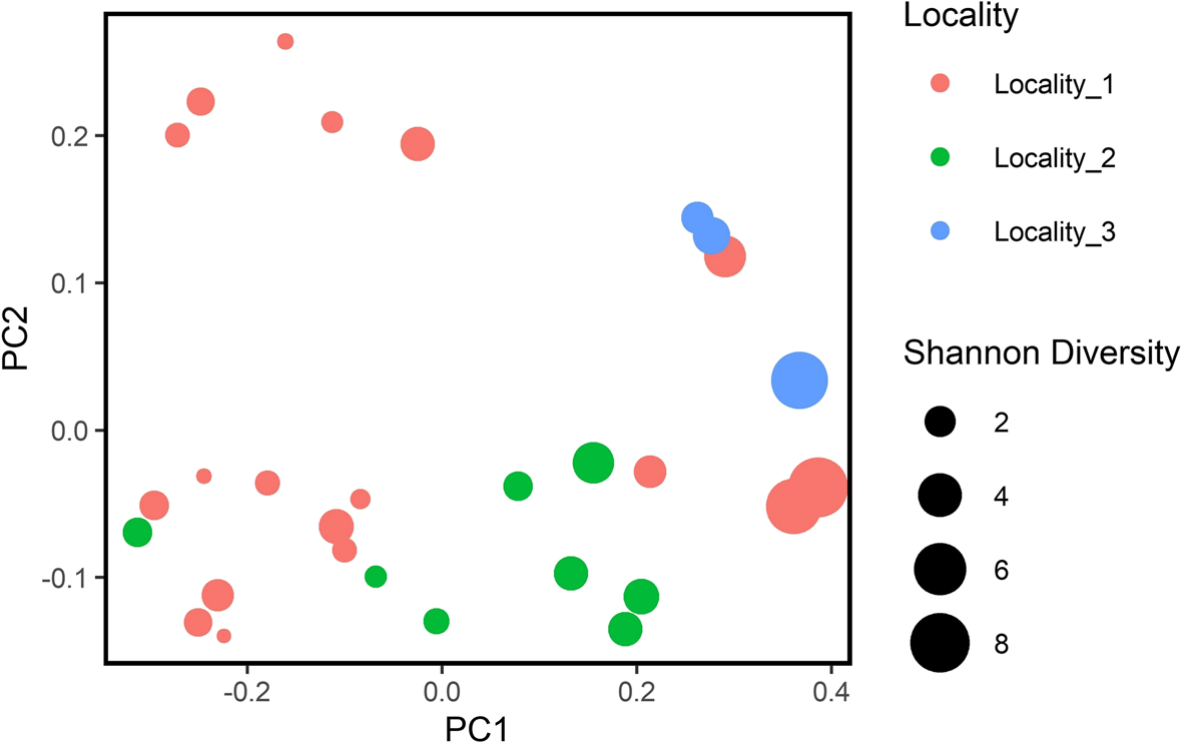
Principal coordinate analysis (PCoA) using unweighted distance data

**Fig. 5:**
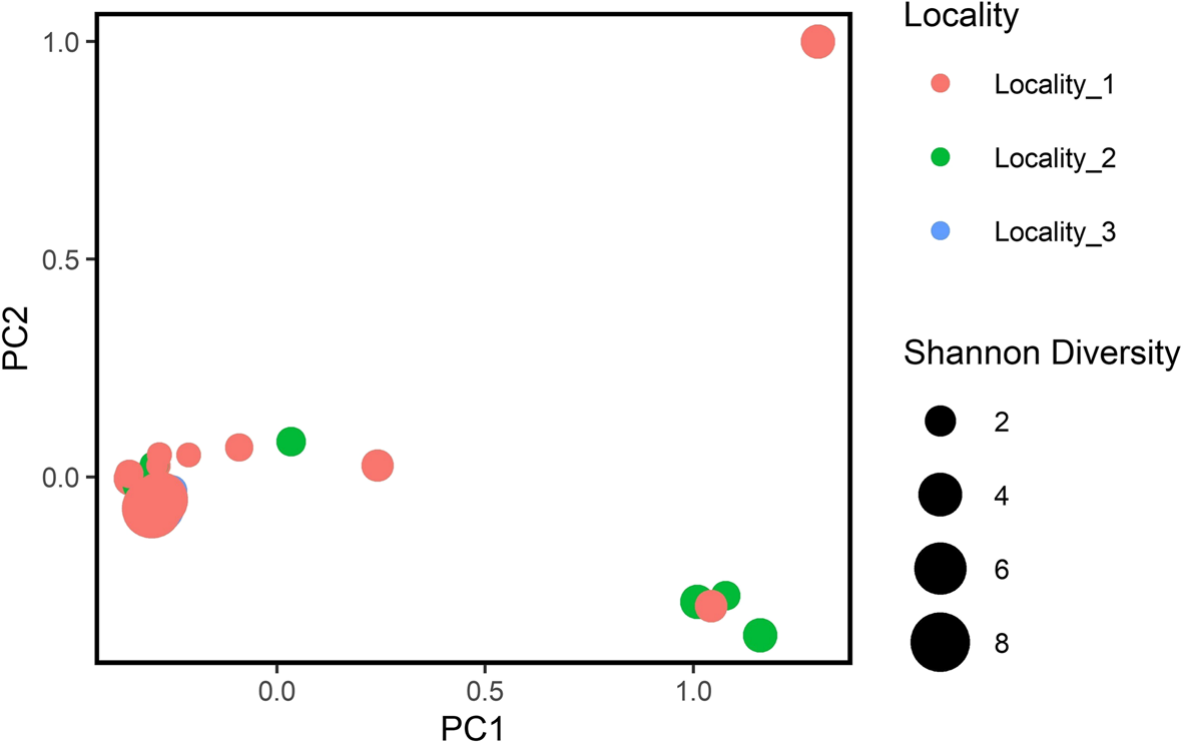
Principal coordinate analysis (PCoA) using weighted distance data

The rarefaction curve showed that the readings in the sequencing were sufficient to infer the abundance of the bacterial community (Supplementary Figs. 1,2,3). All samples were normalized to 48,700 sequences to generate the diversity metrics. This cut-off value was used because it corresponded to the lowest number of contigs found in the sequenced samples as shown in Supplementary Table 2. Although the values of the Shannon–Weaver indices ranged from 1.00 to 8.14 (Supplementary Table 4, Figs. 4 and 5) in the analysed samples, the Kruskal–Wallis test showed no significant difference in the Shannon index in samples from different collection sites ( χ^2^ = 3.146; *p* = 0.207).

The β diversity was calculated in the three groups (Localities #1–#3) and visualized in the PCoAs (Figs. 4 and 5). The results of the PERMANOVA analysis with unweighted Unifrac distance showed significant differences in bacterial composition between samples from Locality #3 and both Localities #1 and #2 (Localities #3 and #1; *p* = 0.012 and Localities #3 and #2; *p* = 0.017). However, no difference was found between the three collection sites using the PERMANOVA analysis with weighted Unifrac distances (*p* > 0.05).

### Differential abundance with ANCOM and heatmap

**Fig. 6:**
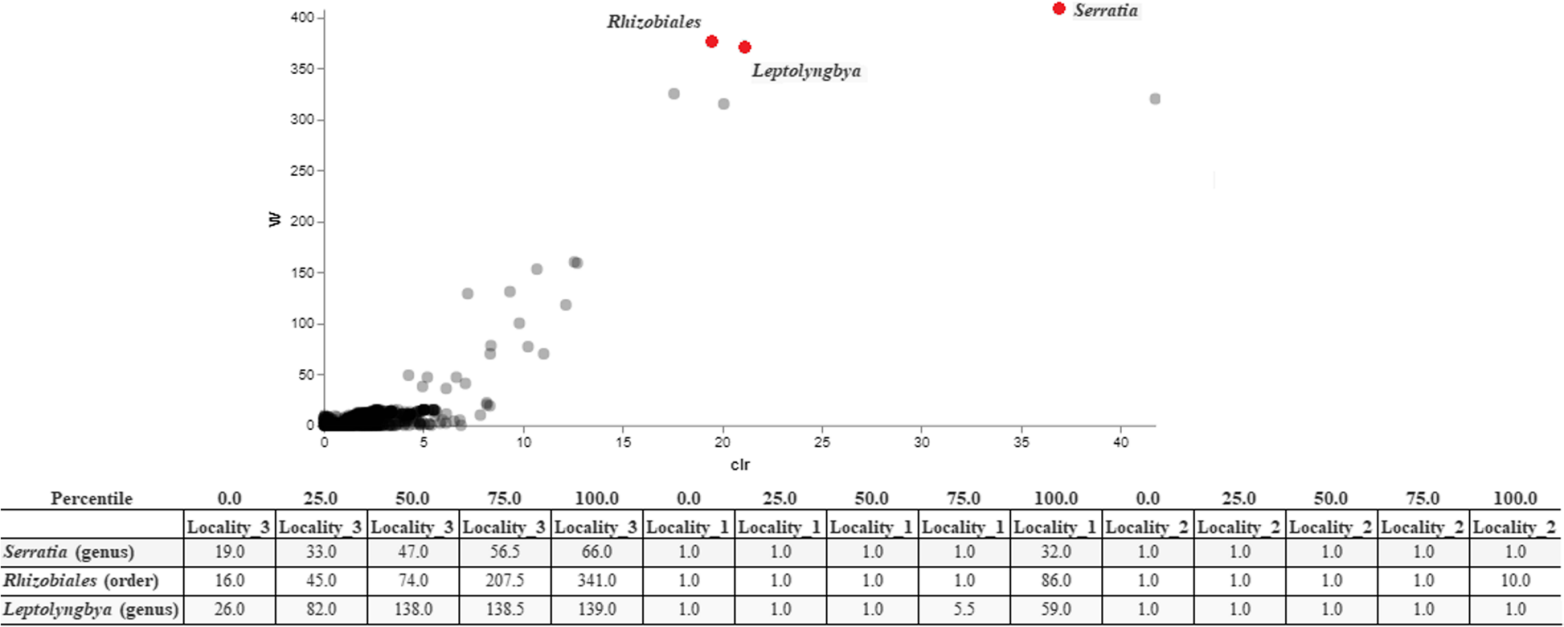
Microbiome composition, the significant ASVs are highlighted

**Fig. 7:**
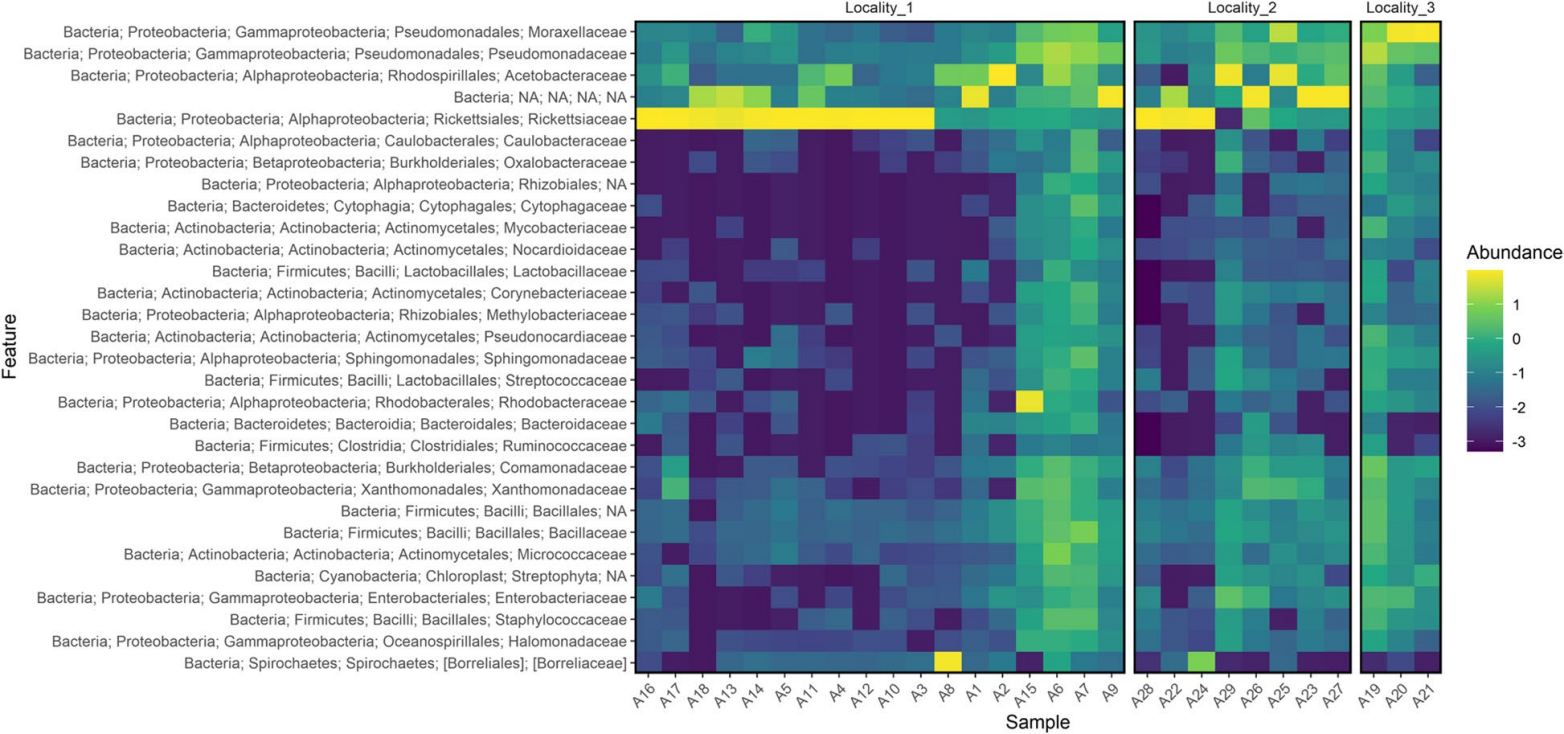
Heatmap of sequences with taxonomic assignment to family nivel

Considering the overall results of the microbiota composition (Fig. 6), it is possible to verify that the *Serratia* and *Leptolyngbya* genera and the *Rhizobiales* order were more abundant in mosquitoes collected from Locality #3. Bacteria of *Rickettsiaceae* family showed the greatest number of sequences in most mosquito females of all three collection sites (Fig. 7).

## Discussion

Despite the importance of *Hg. leucocelaenus* in the transmission cycle of the yellow fever virus in Brazil [36, 47], no investigation focusing on the microbiota associated with this species has been done to date. In addition, the possible role of microbiota in the yellow fever virus mosquito infection remains unknown. The present investigation aimed to verify the bacterial composition and diversity associated with females of *Hg*. *leucocelaenus* and whether these bacterial communities can differ between specimens from different collection sites.

The OTU is an approach widely used in bacterial analyses [15, 48]. Use of an amplicon sequence variant (ASV) instead of an OTU in the microbiota analyses has significant advantages, such as producing an increase in precision and sensitivity for identifying rare organisms [49]. This ASV approach seeks to find unique sequences of each organism instead of performing similarity groupings. In the current study, ASV was chosen in the analyses of the sequences of the V4 region in the 16S gene to increase the likelihood of detecting rare bacteria because the sequence data were generated using NGS in Illumina platform.

*Wolbachia* was identified in most females of *Hg. leucocelaenus* from all three sites in the Vale do Ribeira. This bacterial genus was also found in *Haemagogus* mosquitoes from four forest locations in Trinidad, West Indies and seems to have strong evidence of association with these mosquitoes [50]. Despite the finding that *Wolbachia* is not found naturally in *Ae. aegypti* and anophelines mosquitoes, the artificial introduction of this bacterium into *Ae. aegypti* is one of the control strategies under consideration for elimination of arboviruses transmitted by this mosquito [51]. The biological role of *Wolbachia* in the modulation of arbovirus infection in *Hg. leucocelaenus* needs further investigation, for instance, consisting of females naturally infected with yellow fever virus with noninfected field-collected females. The absence of *Asaia* bacteria in *Hg. leucocelaenus* can be explained by the mutual exclusion that exists between *Wolbachia* and *Asaia*. The exclusion effect was demonstrated in *Anopheles* and *Aedes* mosquitoes [52]. It is highly likely that a similar ecological mechanims is involved in *Hg. leucocelaenus*, but further studies will be needed to confirm the mutual exclusion hypothesis.

The *Wolbachia*, *Swaminathania*, and *Acinetobacter* were the most abundant genera identified in analysed the mosquitoes. The *Swaminathania* genus encompasses nitrogen-fixing bacteria [53], while *Acinetobacter* is involved in blood digestion, playing a role in the parasite-vector interaction in *Ae*. *albopictus* [12, 54]. In the same way, *Acinetobacter* bacteria can be involved in the parasite–vector interaction in *Hg. leucocelaenus*.

The vectorial capacity of a mosquito population involves complex interactions of both biotic and abiotic factors [55]. Despite gaps in the knowledge of interactions between mosquito–microbiota–parasite, it is largely known the influence of the microbiota in the modulation of the vector competence, pathogen extrinsic incubation period and vector density, which are components of the vectorial capacity [5, 56]. In this study, considering unweighted Unifrac distance, the composition of the bacterial communities differed between collection sites and corroborates the results of other studies carried out on *Aedes*, *Anopheles*, and *Culex* [13, 26]. Differences in spatial microbiota composition found in this study provide additional support for the hypothesis that the environment influences the bacterial composition in mosquitoes. Certainly, the effects of the environment and the bacterial composition in the vector and vectorial capacities of *Hg. leucocelaenus* populations need further investigation. In addition, it will be important to verify whether the microbiota can be affected by the season and how the differences can modulate the vectorial capacity of the mosquito vector with potential impact in the transmission dynamics of yellow fever virus.

*Serratia* and *Leptolyngbya* genera and *Rhizobiales* order were found to be more abundant in samples from Locality #3. Bacteria of *Serratia* genus participates in the blood digestion in *Ae*. *aegypti* [57]. Because the amino acids resulting from blood digestion are essential for the mosquito reproductive cycle [58], *Serratia* species can affect fertility in these mosquitoes. In addition, *S. marcescens* modulates the arbovirus infection in *Ae*. *aegypti* via secretion of the SmEnhancin protein [59]. This protein digests mucins that is present in the membrane of the intestinal epithelium of mosquito and disrupts the physical barrier that prevents viral entry, thus faciliting arboviral infection [59]. Some genera of the *Rhizobiales* include bacteria species that fix nitrogen, a process that would affect both the legume nodulators and microsymbiotics [60]. Bacteria of this order were found in field collected adult mosquitoes collected in the state of Missouri (United States) [13] and *An. gambiae* and *Cx. quinquefasciatus* were collected in Kenya. In contrast, *Rhizobiales* bacterias were not detected in laboratory-raised mosquitoes of these species. This finding indicates that the environment may have influenced the bacterial composition in these mosquito species [61].

*Haemagogus leucocelaenus* is a sylvatic primatophile mosquito that is also found in rural and forest fragmented landscapes in the South and Central America [28, 36, 62, 63]. The differences found in bacterial communities of *Hg. leucocelaenus* in the PCoAs analysis were corroborated by the PERMANOVA for which the differences were statistically significant. The biological and ecological meaning of the microbiota differences found in this study needs further investigation, especially to exploit if the differences can affect the processes of yellow fever virus invasion and dissemination and affect the vector and vectorial competence of *Hg. leucocelaenus* along its geographical distribution.

## Conclusions

This study showed significant spatial variation in the bacterial composition of *Hg*. *leucocelaenus* despite the small number of mosquitoes collected in some areas that may have influenced this difference. The presence of *Wolbachia* was recorded in this species and indicates that the vector competence of the species populations can vary along its geographical distribution area. The occurrence of *Serratia* bacterium in *Hg. leucocelaenus* collected from two sites indicates that it was acquired from the environment. The presence of *Serratia* may facilitate viral invasion caused by disruption of the midgut barrier induced by the SmEnhancin protein, which digests the mucins present in the intestinal epithelium.

## Supplementary Information

**Additional file 1: Supplementary Figure 1.** Rarefaction curve showing that the reads in the sequencing were sufficient to infer the abundance of the bacterial community. 

**Additional file 2: Supplementary Figure 2.** Rarefaction curve showing the relation of the reads in the sequencing of each sample and the bacterial Shannon diversity.

**Additional file 3: Supplementary Figure 3. **Rarefaction curve showing the relation of total mosquito of each site and the bacterial Shannon diversity.

**Additional file 4: Supplementary Table 1. **Details of the collections carried out in the localities in Atlantic Tropical Rain Forest, in Vale do Ribeira, São Paulo, Brazil, in 2020. **Supplementary Table 2. **Number of total raw reads and contigs recovered for each female of *Haemagogus leucocelaenus*. **Supplementary Table 4.** Shannon-Weaver index calculated for the bacterial community recovered from each female of *Haemagogus leucocelaenus*.

**Additional file 5: Supplementary Table 3. **Number of contigs of each taxon per sample. 

## Data Availability

Sequence data (individual fastq files) are available from the NCBI Sequence Read Archive under accession PRJNA820596.
