# Supplementary figures and images for: Bacterial diversity in Haemagogus leucocelaenus (Diptera: Culicidae) from Vale do Ribeira, São Paulo, Brazil
Source: BMC Microbiol. 2022 Jun 22;22:161. doi: 10.1186/s12866-022-02571-5 (PMC9215073; doi:10.1186/s12866-022-02571-5)

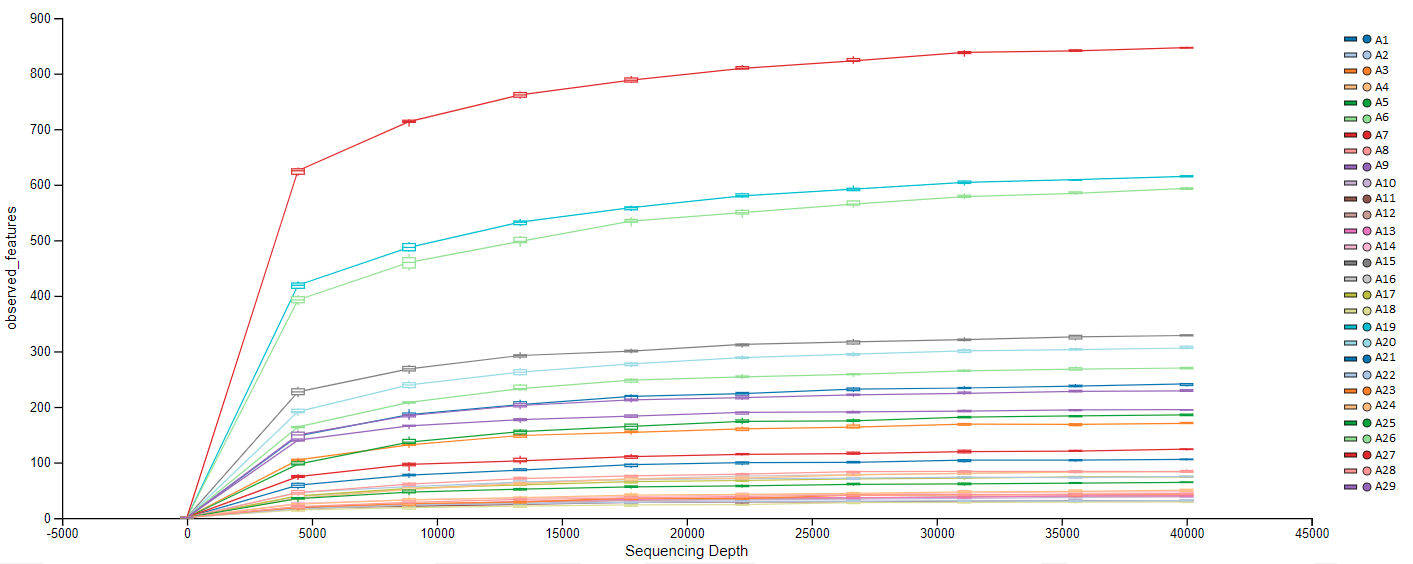

Supplement: Supplementary file 1 — Additional file 1: Supplementary Figure 1. Rarefaction curve showing that the reads in the sequencing were sufficient to infer the abundance of the bacterial community. [file 12866_2022_2571_MOESM1_ESM.png]

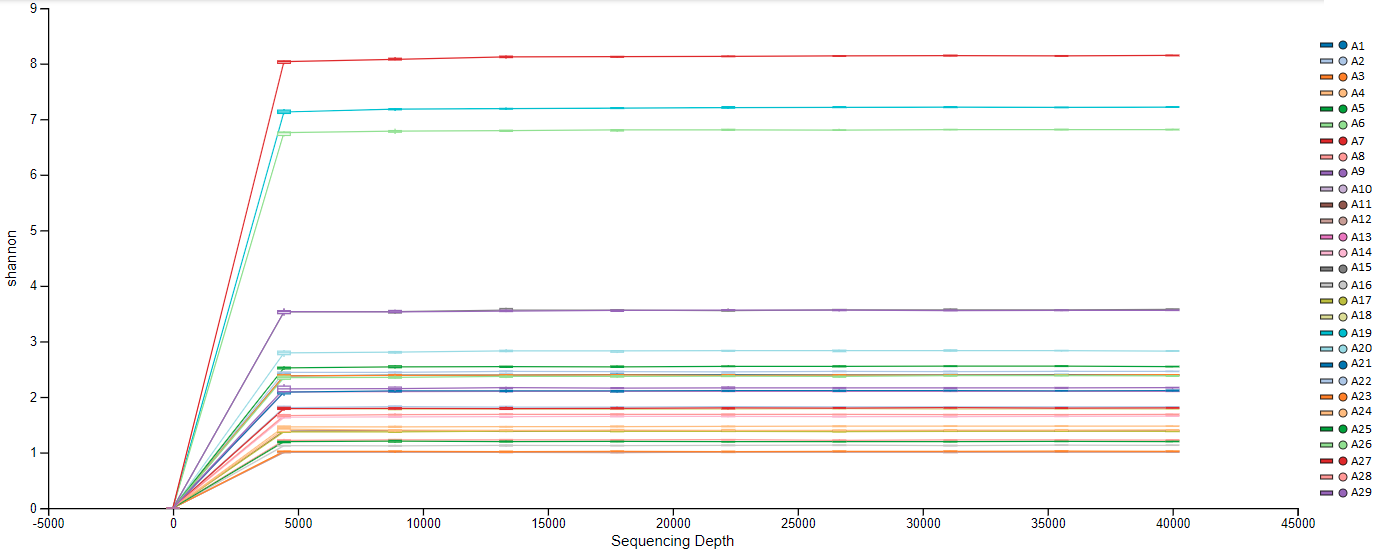

Supplement: Supplementary file 2 — Additional file 2: Supplementary Figure 2. Rarefaction curve showing the relation of the reads in the sequencing of each sample and the bacterial Shannon diversity. [file 12866_2022_2571_MOESM2_ESM.png]

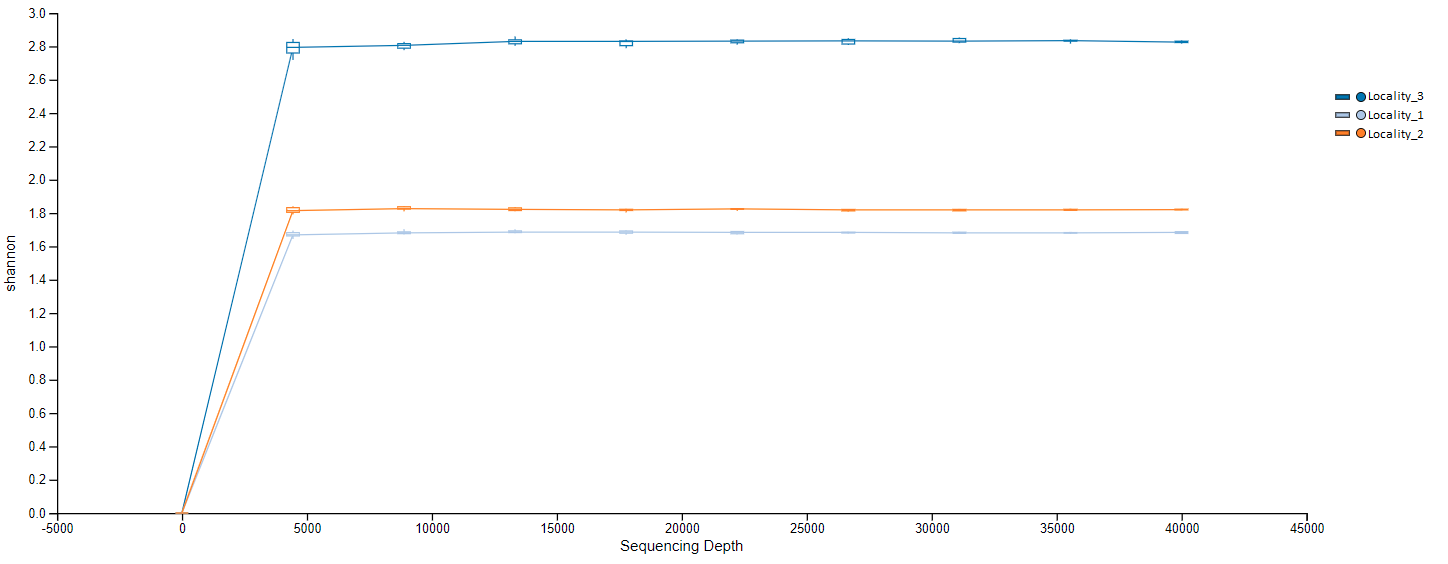

Supplement: Supplementary file 3 — Additional file 3: Supplementary Figure 3. Rarefaction curve showing the relation of total mosquito of each site and the bacterial Shannon diversity. [file 12866_2022_2571_MOESM3_ESM.png]
