# Supplementary material for: Bacterial diversity in Haemagogus leucocelaenus (Diptera: Culicidae) from Vale do Ribeira, São Paulo, Brazil
Source: BMC Microbiol. 2022 Jun 22;22:161. doi: 10.1186/s12866-022-02571-5 (PMC9215073; doi:10.1186/s12866-022-02571-5)
Supplement: Supplementary file 4 — Additional file 4: Supplementary Table 1. Details of the collections carried out in the localities in Atlantic Tropical Rain Forest, in Vale do Ribeira, São Paulo, Brazil, in 2020. Supplementary Table 2. Number of total raw reads and contigs recovered for each female of Haemagogus leucocelaenus. Supplementary Table 4. Shannon-Weaver index calculated for the bacterial community recovered from each female of Haemagogus leucocelaenus. [file 12866_2022_2571_MOESM4_ESM.docx]

**Bacterial diversity in *Haemagogus leucocelaenus* (Diptera: Culicidae) from Vale do Ribeira, São Paulo, Brazil**

**Authors:** Herculano da Silva^1^, Tatiane M. P. Oliveira^1^, Ester C. Sabino^2#^, Diego Peres Alonso^1,3^, Maria Anice M. Sallum^1^

^1^Departamento de Epidemiologia, Faculdade de Saúde Pública, Universidade de São Paulo, Av. Dr. Arnaldo 715, São Paulo, SP 01246-904, Brazil

^2^Departamento de Moléstias Infecciosas e Parasitarias, Instituto de Medicina Tropical da Faculdade de Medicina da Universidade de São Paulo, Av. Dr. Eneas de Carvalho 470, 1º andar, São Paulo, 05403-000 Brazil

^3^Biotechnology Institute and Bioscience Institute, Sao Paulo State University (UNESP), Botucatu, 18618-689, Brazil.

**Supplementary Table 1.** Details of the collections carried out in the localities in Atlantic Tropical Rain Forest, in Vale do Ribeira, São Paulo, Brazil, in 2020.

| State | Municipality | Locality ID | Sample ID | Species ID | Coll Month |
| --- | --- | --- | --- | --- | --- |
| São Paulo | Pariquera Açu | Locality_1 | A1 | *Hg. leucocelaenus* | February |
| São Paulo | Pariquera Açu | Locality_1 | A2 | *Hg. leucocelaenus* | February |
| São Paulo | Pariquera Açu | Locality_1 | A3 | *Hg. leucocelaenus* | February |
| São Paulo | Pariquera Açu | Locality_1 | A4 | *Hg. leucocelaenus* | January |
| São Paulo | Pariquera Açu | Locality_1 | A5 | *Hg. leucocelaenus* | January |
| São Paulo | Pariquera Açu | Locality_1 | A6 | *Hg. leucocelaenus* | February |
| São Paulo | Pariquera Açu | Locality_1 | A7 | *Hg. leucocelaenus* | February |
| São Paulo | Pariquera Açu | Locality_1 | A8 | *Hg. leucocelaenus* | January |
| São Paulo | Pariquera Açu | Locality_1 | A9 | *Hg. leucocelaenus* | January |
| São Paulo | Pariquera Açu | Locality_1 | A10 | *Hg. leucocelaenus* | January |
| São Paulo | Pariquera Açu | Locality_1 | A11 | *Hg. leucocelaenus* | January |
| São Paulo | Pariquera Açu | Locality_1 | A12 | *Hg. leucocelaenus* | January |
| São Paulo | Pariquera Açu | Locality_1 | A13 | *Hg. leucocelaenus* | January |
| São Paulo | Pariquera Açu | Locality_1 | A14 | *Hg. leucocelaenus* | January |
| São Paulo | Pariquera Açu | Locality_1 | A15 | *Hg. leucocelaenus* | January |
| São Paulo | Pariquera Açu | Locality_1 | A16 | *Hg. leucocelaenus* | February |
| São Paulo | Pariquera Açu | Locality_1 | A17 | *Hg. leucocelaenus* | January |
| São Paulo | Pariquera Açu | Locality_1 | A18 | *Hg. leucocelaenus* | January |
| São Paulo | Cananeia | Locality_3 | A19 | *Hg. leucocelaenus* | January |
| São Paulo | Cananeia | Locality_3 | A20 | *Hg. leucocelaenus* | January |
| São Paulo | Cananeia | Locality_3 | A21 | *Hg. leucocelaenus* | January |
| São Paulo | Cananeia | Locality_2 | A22 | *Hg. leucocelaenus* | January |
| São Paulo | Cananeia | Locality_2 | A23 | *Hg. leucocelaenus* | January |
| São Paulo | Cananeia | Locality_2 | A24 | *Hg. leucocelaenus* | January |
| São Paulo | Cananeia | Locality_2 | A25 | *Hg. leucocelaenus* | January |
| São Paulo | Cananeia | Locality_2 | A26 | *Hg. leucocelaenus* | January |
| São Paulo | Cananeia | Locality_2 | A27 | *Hg. leucocelaenus* | January |
| São Paulo | Cananeia | Locality_2 | A28 | *Hg. leucocelaenus* | January |
| São Paulo | Pariquera Açu | Locality_1 | A29 | *Hg. leucocelaenus* | February |

**Supplementary Table 2.** Number of total raw reads and contigs recovered for each female of *Haemagogus leucocelaenus*.

| Sample ID | Forward sequences | Reverse sequences | Contigs generated after FLASH | Contigs after Dada2 plugin |
| --- | --- | --- | --- | --- |
| A1 | 98,804 | 98,804 | 97,256 | 91,265 |
| A2 | 84,224 | 84,224 | 83,172 | 63,902 |
| A3 | 89,956 | 89,956 | 88,816 | 86,624 |
| A4 | 111,293 | 111,293 | 110,344 | 105,871 |
| A5 | 79,442 | 79,442 | 78,581 | 75,949 |
| A6 | 91,950 | 91,950 | 87,201 | 56,441 |
| A7 | 85,202 | 85,202 | 79,239 | 48,727 |
| A8 | 102,007 | 102,007 | 99,164 | 86,967 |
| A9 | 86,324 | 86,324 | 83,973 | 77,432 |
| A10 | 106,838 | 106,838 | 105,761 | 103,478 |
| A11 | 92,506 | 92,506 | 91,306 | 86,975 |
| A12 | 93,081 | 93,081 | 92,108 | 89,631 |
| A13 | 102,418 | 102,418 | 100,719 | 91,742 |
| A14 | 106,528 | 106,528 | 105,235 | 101,339 |
| A15  A16  A17  A18  A19  A20  A21  A22  A23  A24  A25  A26  A27  A28  A29 | 90,551  96,392  91,418  109,338  83,692  80,828  85,650  99,346  102,724  93,342  89,932  107,465  92,847  205,786  85,627 | 90,551  96,392  91,418  109,338  83,692  80,828  85,650  99,346  102,724  93,342  89,932  107,465  92,847  205,786  85,627 | 86,755  95,403  90,091  107,982  77,762  77,827  84,109  87,700  98,973  91,978  88,026  103,679  90,271  202,942  83,618 | 72,850  92,685  85,705  101,080  49,013  66,092  77,043  91,234  77,071  83,218  77,346  70,492  81,989  195,762  54,324 |
|  |  |  |  |  |

**Supplementary Table 4**. Shannon-Weaver index calculated for the bacterial community recovered from each female of *Haemagogus leucocelaenus*.

| Sample |  | Shannon index |  |
| --- | --- | --- | --- |
| A1 |  | 2.39 |  |
| A2 |  | 2.46 |  |
| A3 |  | 1.02 |  |
| A4 |  | 1.40 |  |
| A5 |  | 1.19 |  |
| A6 |  | 6.81 |  |
| A7 |  | 8.14 |  |
| A8 |  | 1.68 |  |
| A9 |  | 2.16 |  |
| A10 |  | 1.00 |  |
| A11 |  | 1.38 |  |
| A12 |  | 1.00 |  |
| A13  A14  A15  A16  A17  A18  A19  A20  A21  A22  A23  A24  A25  A26  A27  A28  A29 |  | 2.10  1.64  3.57  1.12  1.37  1.78  7.22  2.83  2.12  1.81  2.39  1.47  2.55  2.38  1.79  1.22  3.56 | |
